# Assessment of Biosecurity in Poultry Farms in Chitwan, Nepal

**DOI:** 10.1002/vms3.70232

**Published:** 2025-02-06

**Authors:** Alok Dhakal, Sachin Devkota, Sher Bahadur Jethara, Rakesh Kumar Yadav, Parshuram Phuyal

**Affiliations:** ^1^ Paklihawa Campus, Institute of Agriculture and Animal Science Tribhuvan University Bhairahawa Rupandehi Nepal; ^2^ National Avian Disease Investigation Laboratory Bharatpur Nepal

**Keywords:** avian influenza, biosecurity, disease, poultry

## Abstract

The occurrence of poultry disease is one of the major problems for poultry farmers. Proper implementation of biosecurity practices leads to a reduction in entry, occurrence and spread of pathogens on farms, that have negative consequences for animal health, human health and economy. The goal of the study was to assess biosecurity measures implemented by broiler and layer farmers in Chitwan, Nepal. A total of 400 poultry farmers were surveyed using a structured questionnaire. The mean conceptual, structural and operational biosecurity scores obtained by the farms were 4.7 ± 1.2, 11.6 ± 2.7 and 17.1 ± 4.1, respectively. The average biosecurity score recorded was 33.4 ± 6.7. The lowest score obtained by a farm was 7, whereas the highest score obtained was 47. It was found that out of 400 farms, 44.2% (177/400) maintained a satisfactory level of biosecurity, while the remaining 223 (55.8%) exhibited an unsatisfactory level. The chi‐square test revealed that the main occupation (*χ* ^2^ = 31.832, *p* < 0.001), experience in poultry farming (*χ* ^2^ = 13.618, *p* < 0.001), attending poultry farming training (*χ* ^2^ = 23.107, *p* < 0.001), biosecurity training (*χ* ^2^ = 15.331, *p* = 0.002), farm capacity (*χ* ^2^ = 41.794, *p* < 0.001), farm type (*χ* ^2^ = 25.002, *p* < 0.001), flooring system (*χ* ^2^ = 35.906, *p* < 0.001) and presence of workers in the farm (*χ* ^2^ = 44.024, *p* < 0.001) were significantly associated with the biosecurity level in poultry farms. This study reveals that there is much space for improvement in the adoption of biosecurity measures by poultry farms. Future training programs for poultry farmers should focus on providing knowledge on the proper implementation of biosecurity measures as a strategy for disease prevention and control.

## Graphical abstract

The study aimed to evaluate biosecurity practices of broiler and layer farmers in Chitwan, Nepal. A total of 400 poultry farmers participated in the survey, which was conducted using a structured questionnaire. The average scores for conceptual, structural and operational biosecurity were 4.7 ± 1.2, 11.6 ± 2.7 and 17.1 ± 4.1, respectively. The overall average biosecurity score was 33.4 ± 6.7. The scores ranged from a minimum of 7 to a maximum of 47. The study found that 177 farms (44.2%) maintained a satisfactory level of biosecurity, while the remaining 223 farms (55.8%) had an unsatisfactory level of biosecurity.

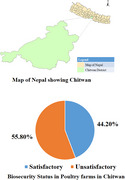

## 1 Introduction

Poultry and its products provide a significant source of animal protein for human consumption in Nepal and all over the world. According to MoALD (2023), around 66 million fowl are thought to be present in Nepal as of 2021–2022. In recent years, the Nepalese poultry industry has witnessed a significant transition in terms of investment size, operational expenses, marketing approaches, employment opportunities and revenue creation (Sharma 2010). This sector has played a vital role in meeting the ever‐increasing demand for protein‐rich food and contributing to the development of the economy in Nepal, accounting for about 3.5% of total GDP (Dhakal et al. 2019).

Biosecurity on farms refers to a comprehensive set of actions and measures used to reduce the possibility of introduction and spread of disease on the farm (Siekkinen et al. 2012). It has been accepted as one of the most cost‐effective methods for controlling poultry diseases and preventing zoonotic disease transmission in humans (Gelaude et al. 2014). It is evident that the losses created by disease outbreaks are significantly more expensive than what it would cost to properly adopt biosecurity measures (Tanquilut et al. 2020). Animal health, technical and financial performance, as well as the low usage of antimicrobials, are all enhanced in animal production when the biosecurity level is high (Dewulf and Immerseel 2019).

Various researchers have highlighted that a higher incidence and outbreak of avian diseases are linked to lower levels of biosecurity in farms (Aila et al. 2012; Van Limbergen et al. 2018). For example, many economically important poultry diseases, including Highly pathogenic avian influenza (HPAI) (Garber et al. 2016; Gonzales et al. 2017), Newcastle disease (ND) (Wiseman et al. 2018), Infectious laryngotracheitis (ILT) (Volkova et al. 2012) and Salmonellosis (Snow et al. 2010), have been linked to poor biosecurity practices. The periodic outbreak of avian diseases like Avian influenza (AI) in Nepal has created significant losses for the growing poultry industry (Gompo et al. 2020). Sharma (2010) states that poultry farms in Nepal lack proper adoption of biosecurity measures. This fact is supported by Gompo et al. (2020), who affirm that the majority of small‐ and medium‐scale Nepalese poultry farmers maintain a subpar level of biosecurity. Thus, the appearance and spread of different poultry diseases continually undermine the expansion and sustainability of poultry sector. As a result, it causes substantial financial losses, decreased profitability, a loss of interest in chicken farming and, in some situations, poses a zoonotic threat to farm employees.

As biosecurity measures are essential for preventing disease entry and spread, increasing poultry performance and enhancing the quality of chicken production, there is a need to assess the biosecurity measures adopted in poultry farms in Nepal. So, the study was conducted in Chitwan district, which is one of the poultry hubs in Nepal. The objective of the study was to examine the biosecurity measures employed by poultry farms in Chitwan, Nepal. This study can help in the development of strategies and policies to prevent and control the spread of diseases by identifying gaps in implementation of biosecurity measures on poultry farms.

## 2 Methodology

### 2.1 Ethical Approval

The study was approved by the Nepal Veterinary Council (Ref. No. Ethical 30/2080/81).

### 2.2 Study Area

**FIGURE 1:**
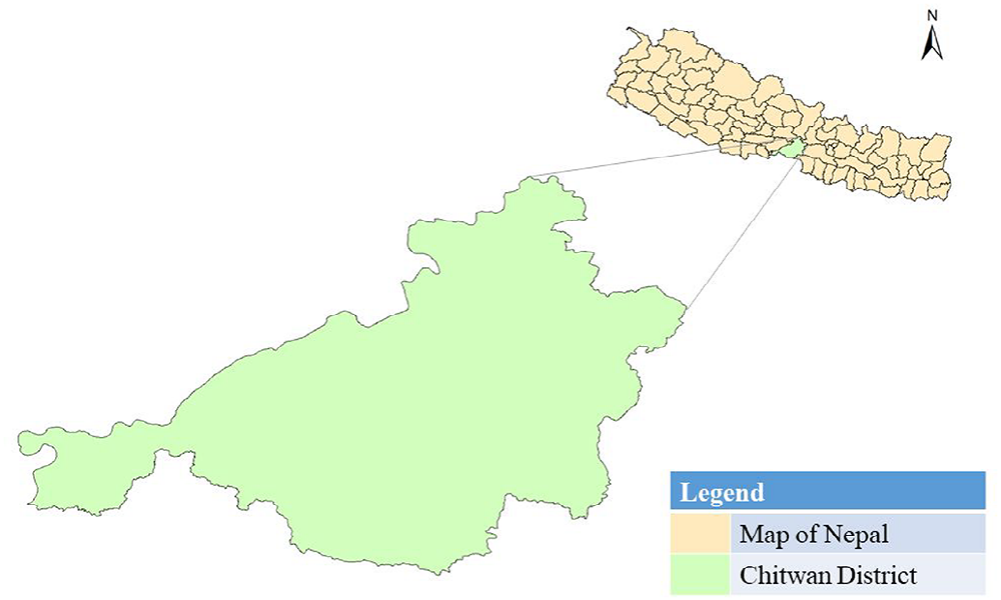
Map of Nepal showing Chitwan district. The shape file for ArcGIS Version 10.8 was obtained from https://nationalgeoportal.gov.np.

The study was carried out in Chitwan district, Nepal. It was selected purposively as it is known as the poultry hub of Nepal. Chitwan district is located in Bagmati province and lies in the Terai plains of Nepal. According to MoALD (2023), in fiscal year 2021–2022, Chitwan district had a total number of 6,722,623 fowl, which was around 50% of total poultry in Bagmati Province. The map of Nepal showing the Chitwan district is presented in Figure 1.

### 2.3 Sample Size and Sampling Procedure

A total of 400 poultry farms were surveyed, that is, 5% more than the required sample size of 385. It (the sample size required: 385) was determined with a 95% confidence level, a 5% margin of error and a population proportion of 50% using the online sample size calculator portal (https://www.calculator.net/sample‐size‐calculator.html). By assuming 50%, we tried to minimize the risk of underestimating the required sample size, which could lead to greater sampling error and less reliable results. Owing to a lack of information on details of poultry farms, the snowball sampling method was used, where a respondent from one farm provided information about the next nearby poultry farm to be interviewed. This was followed until the target number of respondents was achieved.

### 2.4 Data Collection Method

The questionnaire was prepared by researchers with the help of similar studies carried out (Ismael et al. 2021; Tsegaye et al. 2023). Pre‐testing of the questionnaire was done with 20 poultry farmers to ensure that the questions were clear and could effectively capture the intended objectives. The modified questionnaire was finalized for data collection, incorporating inputs received and changes required. The response from the pre‐test was not incorporated into the final dataset. The questionnaire was divided into three main sections. In the first section, farm characteristics were accessed. It was then followed by biosecurity accessing questions, which were classified into conceptual biosecurity (7 questions), structural biosecurity (16 questions) and operational biosecurity (27 questions). The last section had questions relating socio‐demographics of the respondent and owner of farm. A face‐to‐face interview was carried out using questionnaires that were set in the KoboToolbox and read by the investigator in Nepali (the local language). The respondents were made aware of the purpose of the study, their verbal consent was obtained and privacy was maintained. Those who refused to participate were not included in the study.

### 2.5 Data Analysis

After completion of data collection, the Excel file from KoboToolbox was imported to IBM Statistical Package for Social Sciences (SPSS) Version 20. The data was checked for incompleteness and missing values. Each farm was assessed based on various biosecurity indicators, with a score of 1 assigned for each correctly implemented practice and score of zero corresponding to no implementation of a biosecurity measure, as done by Maduka et al. (2016). The final biosecurity score (BS) for each farm was determined by calculating the sum of scores across all indicators. The maximum score that a farm could score was 50. If a farm could get a BS score of ≥ 35 (70% of total BS), then it was categorized as a farm with satisfactory biosecurity and others as a farm with unsatisfactory biosecurity practices. To check for association between the dependent and independent variables, Chi‐square test was used. A *p* < 0.05 was judged statistically significant.

## 3 Results

### 3.1 Demographic Profile

Most of the respondents (95%) were owners of poultry farms. The average age of respondents was 41 years. It was found that farm owners had an average of 10 years of education and 7.5 years of experience in raising poultry. Around 20% of owners were engaged in other occupations rather than poultry farming as their primary occupation. Seven in 10 respondents received poultry farming training, while only 45% received biosecurity‐related training (Table 1).

**TABLE 1:** Demographic details of respondents and owners in the study area.

Demographic details	Frequency (%)
Are you owner of the farm?	
No	20 (5%)
Yes	380 (95%)
Gender of respondent?	
Female	59 (14.8%)
Male	341 (85.2%)
Primary occupation of owner?	
Others	83 (20.8%)
Poultry	317 (79.2%)
Have you (or owner) attended any training on poultry farming?	
No	122 (30.5%)
Yes	278 (69.5%)
Have you (or owner) obtained training on biosecurity?	
No	220 (55%)
Yes	180 (45%)

### 3.2 Farm Characteristics

On average, a poultry farm could accommodate approximately 2505 poultry. Most of the poultry farmers had their own farm premises (94.2%, 377/400). Commercial broilers were the predominant poultry species reared in study area. It was found that 9 in 10 people used commercial feed for poultry, while 1 in 10 prepared the poultry feed themselves. More than half of farms had farm workers on their farms. The characteristics of farm in the study area are shown in Table 2.

**TABLE 2:** Farm characteristics in the study area.

Farm characteristics	Frequency (%)
Farm premises	
Rented	23 (5.8%)
Owned	377 (94.2%)
Bird type	
Broilers	214 (53.5%)
Layers	169 (42.3%)
Both broilers and layers	17 (4.2%)
Feed type	
Self	43 (10.8%)
Commercial	357 (89.2%)
Flooring system	
Cemented	341 (85.2%)
Muddy	55 (13.8%)
Wooden	2 (0.5%)
Others	2 (0.5%)
Presence of worker	
Yes	215 (53.7%)
No	185 (46.3%)

### 3.3 Biosecurity Component

#### 3.3.1 Conceptual Biosecurity

Different indicators of conceptual biosecurity are presented in Table 3. Half (50.5%, 202/400) of the farms were located close to the main road, that is, ≤ 200 m from the main road. Furthermore, 6 out of 10 farms were more than 1 km from the nearest farm. Most (85.5%, 342/400) of the poultry farms in the study area were oriented in east–west direction. We found that 9 out of 10 farms did not allow visitors to enter poultry compartments.

**TABLE 3:** Indicators of conceptual biosecurity.

Indicators of conceptual biosecurity	Frequency (%)
How far is the distance of the farm from the main road (m)?	
≤ 200 m	202 (50.5%)
≥ 201 m	198 (49.5%)
How far is the distance of the farm from the nearest farm (km)?	
≤ 1 km	164 (41.0%)
≥ 1.1 km	236 (59.0%)
How far is the distance of the farm from the residential place (m)?	
≤ 500 m	312 (78.0%)
≥ 501 m	88 (22.0%)
Is there presence of stagnant water in the farm?	
Yes	110 (27.5%)
No	290 (72.5%)
To which direction is the poultry house oriented?	
East–West	342 (85.5%)
Others	58 (14.5%)
Is the poultry house provided with good ventilation?	
Yes	376 (94.0%)
No	24 (6.0%)
Do visitors have access to poultry compartment?	
Yes	31 (7.8%)
No	369 (92.3%)

#### 3.3.2 Structural Biosecurity

From the chicken farms assessed, 57.5% (230/400) had a fence and gate and 88.5% (354/400) had a footbath at their farm (Table 4). Results indicated that only 56% (224/400) of farms kept ‘No entry without permission’ or ‘Biosecurity’ signage on their farms. Similarly, no farms were found using surface water for drinking purposes for chickens. Nearly 2 in 10 poultry farms had pets on their farms. Few farmers had the habit of exchanging equipment with other farms. Similarly, it was found that 61.5% (246/400) of farms had a proper quarantine or either an isolation room.

**TABLE 4:** Structural biosecurity indicators.

Indicators of structural biosecurity	No	Yes
Is there a fence and gate on the farm?	170 (42.5%)	230 (57.5%)
Is there a footbath at the farm gate?	46 (11.5%)	354 (88.5%)
Are chicken houses constructed of impervious material?	54 (13.5%)	346 (86.5%)
Is there signage board at the farm? (no entry without permission or biosecurity board)	176 (44.0%)	224 (56.0%)
Are there other animals in the farm?	335 (83.8%)	65 (16.2%)
Is there only one vehicle entry point?	59 (14.8%)	341 (85.3%)
Do you exchange equipment with other farm?	394 (98.5%)	6 (1.5%)
Is surface water (e.g., streams, rivers, etc.) used for drinking purpose for poultry?	400 (100%)	0
Is there a pet animal in the farm?	330 (82.5%)	70 (17.5%)
Is there permanent rodent control program in the farm?	287 (71.8%)	113 (28.2%)
Is there a proper feed store?	60 (15.0%)	340 (85.0%)
Is poultry area accessible to wild birds?	197 (49.3%)	203 (50.7%)
Do wild bird has access to stored food?	320 (80.0%)	80 (20.0%)
Do wild bird has access to stored fresh litter?	226 (56.5%)	174 (43.5%)
Do you stay informed regarding the disease outbreaks in the area?	143 (35.8%)	257 (64.3%)
Do you have a quarantine or isolation room for sick animal/new incoming flock?	154 (38.5%)	246 (61.5%)

#### 3.3.3 Operational Biosecurity

As indicated in Table 5, it can be observed that on 48.3% (193/400) farms, farm workers or owners did not use specialized cloth. Similarly, 13.5% (54/400) did not use specific footwear during farm work. In addition, 75% (300/400) did not engage in regular cleaning of caps and coveralls. We found that 81.5% (326/400) had practice of keeping few days gap before entering a new batch of chicks. Nearly 95% (379/400) of farms vaccinated their chickens according to their age. Eight in 10 farms regularly disinfected drinking water. Other operational biosecurity indicators are presented in Table 5.

**TABLE 5:** Indicators of operational biosecurity.

Indicators of operational biosecurity	No	Yes
Do farm workers/you use special cloth?	193 (48.3%)	207 (51.7%)
Do farm workers/you use special footwear?	54 (13.5%)	346 (86.5%)
Do farm workers/you use special masks?	57 (14.2%)	343 (85.8%)
Do farm workers/you use special hats?	320 (80.0%)	80 (20.0%)
Do farm workers/you take shower when in and out of farm?	234 (58.5%)	166 (41.5%)
Is there regular laundering of hats and coveralls?	300 (75.0%)	100 (25.0%)
Do visitors wear special cloth?	382 (95.5%)	18 (4.5%)
Do visitors wear special footwear?	248 (62.0%)	152 (38.0%)
Do you prohibit vehicle entry to farm?	118 (29.5%)	282 (70.5%)
Do you keep a visitor logbook?	356 (89.0%)	44 (11.0%)
Do you follow ‘All in all‐out’ system?	27 (6.8%)	373 (93.3%)
Is there regular cleaning and disinfection of the premises?	94 (23.5%)	306 (76.5%)
Is there proper disposal of dead birds?	24 (6.0%)	376 (94.0%)
Do farm workers/ you have contact with another farm?	365 (91.3%)	35 (8.8%)
Is removed litter stored at cover shed?	173 (43.3%)	227 (56.8%)
Do you use high‐pressure sprayer for cleaning?	209 (52.3%)	191 (47.8%)
Do you allow days' gap before entering new lots of chicks?	74 (18.5%)	326 (81.5%)
Do you apply insecticide on to top of new litter?	100 (25.0%)	300 (75.0%)
Is stored feed accessible to rodents?	175 (43.8%)	225 (56.3%)
Do sick birds get examined regularly?	107 (26.8%)	293 (73.3%)
Is there regular sero‐monitoring of chickens?	170 (42.5%)	230 (57.5%)
Do you vaccinate chickens according to age?	21 (5.3%)	379 (94.8%)
Do you use antibiotics when only birds are sick?	31 (7.8%)	369 (92.3%)
Do you use antibiotics based on the recommended dosage by veterinarian?	24 (6.0%)	376 (94.0%)
Do you keep records on the farm? (in‐out, treatment given, vaccine, etc.)	197 (49.3%)	203 (50.7%)
Is spilled feed cleaned up immediately?	125 (31.3%)	275 (68.8%)
Is drinking water regularly disinfected while use?	81 (20.3%)	319 (79.8%)

### 3.4 Factors Affecting Biosecurity Level

The mean BS obtained by the farm was 33.4 ± 6.7. The minimum score that a farm obtained was 7, while a maximum of 47 was obtained. We found that 177 (44.2%) farms had satisfactory biosecurity practices. Similarly, 223 (55.8%) farms had an unsatisfactory biosecurity level. The chi‐square test revealed that main occupation (*χ* ^2^ = 31.832, *p* < 0.001), experience in poultry farming (*χ* ^2^ = 13.618, *p* < 0.001), attending poultry farming training (*χ* ^2^ = 23.107, *p* < 0.001), biosecurity training (*χ* ^2^ = 15.331, *p* = 0.002), farm capacity (*χ* ^2^ = 41.794, *p* < 0.001), farm type (*χ* ^2^ = 25.002, *p* < 0.001), flooring system (*χ* ^2^ = 35.906, *p* < 0.001) and presence of workers in the farm (*χ* ^2^ = 44.024, *p* < 0.001) were found to be significantly associated with the biosecurity status of the farm (Table 6).

**TABLE 6:** Association between biosecurity level and different independent variables.

Variables	Categories	Biosecurity status		*χ* ^2^	df	*p* value
		Unsatisfactory	Satisfactory			
Farm ownership	Male owned	171	147	2.456	1	0.117
	Female owned	52	30			
Age of owner	≤ 40 years	120	83	1.890	1	0.169
	≥ 41 years	103	94			
Owners education level	Basic level (1–8)	57	41	2.024	2	0.364
	Secondary level (9–12)	139	121			
	University level	27	15			
Main Occupation	Poultry	154	163	31.832	1	**<** **0.001**
	Others	69	14			
Experience in rearing poultry	≤ 2 years	36	8	13.618	1	**<** **0.001**
	≥ 3 years	187	169			
Attended training related to poultry farming	Yes	133	145	23.107	1	**<** **0.001**
	No	90	32			
Attended training related to biosecurity	Yes	81	99	15.331	1	**<** **0.001**
	No	142	78			
Poultry premises	Owned	207	70	1.888	1	0.169
	Rented	16	7			
Farm capacity	≤ 1000 birds	90	20	41.794	1	**<** **0.001**
	≥ 1001 birds	133	157			
Farm type	Broiler only	144	70	25.002	2	**<** **0.001**
	Layers only	71	98			
	Both broilers and layers	8	9			
Feed type	Self	18	25	3.768	1	0.052
	Commercial	205	152			
Presence of worker	Yes	87	128	44.024	1	**<** **0.001**
	No	136	49			
Flooring system	Cemented	169	172	35.906	1	**<** **0.001**
	Others	54	5			

Abbreviations: df, degrees of freedom; *χ* ^2^, Pearson's chi‐square value.

## 4 Discussion

This study provides insights on biosecurity measures that are adopted by poultry farms in Chitwan, Nepal. Paudel et al. (2013) state that the risk of AI transmission is intensified by a lack of knowledge and insufficient biosecurity regulation enforcement on poultry farms. Standard biosecurity procedures are significant in reducing outbreaks of different infectious diseases (Gibbens et al. 2001; Halvorson 2011).

The majority of poultry farm owners were male which was also reported by Dhakal and Gompo (2021). Poultry management and biosecurity training should be conducted on a regular basis to farmers. It will improve poultry health, minimize loss and subsequently enhance public health. According to Gelaude et al. (2014), to limit the airborne transmission of diseases from poultry and animals transported along public highways and between poultry farms, the distance to the nearest poultry farm should be at least 500 m and ideally larger than 1 km. Having poultry farms near residential areas provides a biosecurity risk as well as issues with animal and public health due to water, soil and air pollution (Akanni and Benson 2014). In the tropics, the orientation of the farm should be east–west as this helps to minimize exposure to direct sun. But, in temperate regions, it can be constructed to face the rising sun to gain heat inside the farm. Stagnant water and having nearby ponds, lakes and rivers pose a threat of disease transmission to poultry since birds are drawn to these water areas. Different harmful microorganisms are carried by migratory birds, either biologically or mechanically, on the farm (Tawakol et al. 2023). Lambrou et al. (2020) found that majority of farmers (54%) likewise forbade tourists from entering their farms. From this, we can figure out that the farmers are well aware of the risks of permitting guests in poultry compartments. One of the major modes of disease transmission in poultry is the movement of people and animals on and off the farm (Melo et al. 2020).

Similar to our finding, Dhakal and Gompo (2021) found that 53.6% of poultry farms in Chitwan and 69.6% of farms in Kathmandu (69.6%) had fences and gates. Similarly, we found that nearly 90% of the farms in the study district had footbaths at the gate, which was a larger percentage than reported by Dhakal and Gompo (2021). Disinfection footbaths were present at the entrance of 32% of the commercial poultry farms in the study of Lambrou et al. (2020). The application of this measure can be attributed to the farm owner's increased knowledge of diseases spread by the footwear of guests and farm workers. Dhakal and Gompo (2021) found that farms in Chitwan have more quarantine rooms built within them (87.5%) than those in Kathmandu Valley (73.2%). Similarly, Dhakal and Gompo (2021) reported that both Chitwan (46.4%) and Kathmandu (53.6%) had a high percentage of farms having interaction with wild birds, while only an average of 24.2% of farms had contact with nearby wild animals. A proper feed storage room should be an essential requirement while constructing a farm. Poor storage of feed can lead to contamination of feed with *Salmonella* spp., *Escherichia coli*, *Clostridium* spp., *Aspergillus* spp. and mycotoxins. Feed contamination can also happen at various points during the production, storage or transportation of feed (Ismael et al. 2021).

Wild birds and rodents should not have access to feed and litter, as these can spread pathogenic microorganisms that have a significant negative impact on poultry producers (Ismael et al. 2021). In particular, when migratory wild birds have access to feed, they pose a risk as they are the primary source of transboundary disease transmission. It is important that rodent entry be controlled and kept to a minimum for good biosecurity maintenance. Scott et al. (2018) reported in their study that all sorts of farms had dogs, cats and ruminants. One of the reasons to keep these pets is their aid in rodent management and preventing the entry of wild animals. The possibility that their pets would consume improperly disposed dead carcasses is another matter of concern, indicating bad biosecurity implications. So, it is risky to keep these pet animals on farm premises. Moreover, for example, cats have the ability to act as carriers of severe strains of infections caused by *Pasteurella multocida* and *Toxoplasma gondii* in chickens (Beltrame et al. 2012; Sambeek et al. 1995).

Lambrou et al. (2020) in Chitwan found that the majority of farmers (71%) never changed their clothes before or after going to the farm or while handling poultry. The majority of farmers (81%) did not use gloves, 60% did not employ boots and 94% did not wear aprons when interacting with poultry. Similarly, Lambrou et al. (2020) reported that boots and masks were the personal protective equipment (PPE) that were most often worn, whereas aprons and gloves were the PPE that was least used. The study by Hafez et al. (2010) found that poultry farm workers were crucial in the transmission of pathogens from backyard chickens to commercial poultry farms and vice versa. So, workers and owners should be provided with clothes, sandals, masks and hats to use on farm premises. Dhakal and Gompo (2021) reported that the all‐in‐all‐out program was adopted by 91.1% of farmers, the same as our finding of 93.3% of farms practising it. It is important because it is uncertain what diseases the new birds will carry, so they pose a serious threat to biosecurity (Sharma 2010). When disposing dead chickens, it is important to take into account potential consequences on human health, disease transmission and ecological safety. This is because poultry carcasses may have been exposed to highly dangerous AI and other diseases (Hu et al. 2017). Diseases can spread if contaminated birds, equipment or materials are introduced into farms (Tanquilut et al. 2020). Seven in 10 farms prohibited the entry of vehicles into the farm. Letting the vehicles carrying feed or poultry without tire spray or baths poses a serious risk to the farm because these vehicles may transmit harmful microbes across the farm. The farm should only have one entry to reduce the possibility of human mobility, and visitors should sign a log book to help identify people quickly if any disease outbreak events occur. Proper disinfection of farm premises should be done to kill the contagious organisms like *Salmonella* spp. and *Eimeria* spp.

Raising farmer's knowledge on negative impact of antimicrobial misuse and encouraging appropriate biosecurity, farming practices and immunization programs are crucial in low and middle‐income countries to minimize the overuse of antibiotics (Luu et al. 2021). Similarly, Ornelas‐Eusebio et al. (2020) also stated that antimicrobial use can be reduced by raising awareness of biosecurity management and proper farm practices. For example, giving farmers proper information and training on biosecurity management led to a 29% reduction in antimicrobial use, as evidenced by lower treatment incidences (Gelaude et al. 2014).

Similar to this study, various researchers found a significant association between various demographics of owners and farm characteristics and the biosecurity level of the farm. For example, Tsegaye et al. (2023) found that poultry rearing experience, biosecurity training, having an isolation room, practising proper disposal of dead birds and owing disease log books have statistically significant associations with the biosecurity level of farm. Contradictory to our result, Dorea et al. (2010) found that the flock size did not alter the standard of biosecurity measures used. This may be because biosecurity was widely understood and applied to both small‐ and large‐scale farms in that study area. However, according to other investigations supporting our findings, farmers with larger farm spaces and flock sizes were likely to adopt better biosecurity (Akintunde and Adeoti 2014). Similarly, Ismael et al. (2021) found that occupation, prior training, farm ownership premises and farm capacity were significantly associated with the level of biosecurity on the farm.

This study has some limitations as the data were collected using the snowball sampling technique, which may cause sampling bias.

## 5 Conclusion

The biosecurity practices in poultry farms across Chitwan are notably lacking, with our survey revealing that over half of the farms are not adhering to proper biosecurity protocols. This situation underscores a significant gap in the knowledge and implementation of critical biosecurity measures, which are essential for preventing disease outbreaks and ensuring the health of poultry populations. To address this, there is an urgent need for comprehensive capacity‐building initiatives that focus on educating farmers about the importance of biosecurity. These efforts should include the widespread dissemination of information, tailored training programs and ongoing awareness campaigns aimed at equipping farmers with the necessary skills and knowledge to implement effective biosecurity practices. By fostering a culture of biosecurity among farmers, the sector can become more self‐sustaining, better protected against disease threats and ultimately more profitable. These programs should be collaborative efforts involving government agencies, agricultural extension services and industry stakeholders to ensure that the importance of biosecurity is well understood and consistently applied across the region.

## Author Contributions

**Alok Dhakal**: conceptualization, investigation, writing–original draft, methodology, validation, visualization, writing–review and editing, software, formal analysis, project administration, resources, supervision, data curation. **Sachin Devkota**: resources, supervision, data curation, software, formal analysis, project administration, validation, visualization, writing–review and editing, methodology, investigation, writing–original draft. **Sher Bahadur Jethara**: investigation, writing–original draft, methodology, validation, visualization, writing–review and editing, software, formal analysis, project administration, resources, supervision, data curation. **Rakesh Kumar Yadav**: conceptualization, writing–original draft, methodology, writing–review and editing, software, formal analysis, resources, supervision. **Parshuram Phuyal**: conceptualization, writing–original draft, methodology, validation, visualization, writing–review and editing, software, formal analysis, project administration, data curation, supervision, resources.

## Ethics Statement

The study was approved by the Nepal Veterinary Council (Ref. No. Ethical 30/2080/81).

## Consent

Individual's consent was received before collecting data.

## Conflicts of Interest

The authors declare no conflicts of interest.

## Data Availability

The data used to support the findings of this study are available from the corresponding author upon reasonable request.
